# Effects of Organotins on Crustaceans: Update and Perspectives

**DOI:** 10.3389/fendo.2018.00065

**Published:** 2018-02-27

**Authors:** Éverton L. Vogt, Jorge F. A. Model, Anapaula S. Vinagre

**Affiliations:** ^1^Laboratório de Metabolismo e Endocrinologia Comparada (LAMEC), Departamento de Fisiologia, Federal University of Rio Grande do Sul (UFRGS), Porto Alegre, Brazil

**Keywords:** crustaceans, organotins, endocrine disruption, growth, metabolism, reproduction

## Abstract

Organotins (OTs) are considered some of the most toxic chemicals introduced into aquatic environments by anthropogenic activities. They are widely used for agricultural and industrial purposes and as antifouling additives on boat hull’s paints. Even though the use of OTs was banned in 2008, elevated levels of OTs can still be detected in aquatic environments. OTs’ deleterious effects upon wildlife and experimental animals are well documented and include endocrine disruption, immunotoxicity, neurotoxicity, genotoxicity, and metabolic dysfunction. Crustaceans are key members of zooplankton and benthic communities and have vital roles in food chains, so the endocrine-disrupting effects of tributyltin (TBT) on crustaceans can affect other organisms. TBT can disrupt carbohydrate and lipid homeostasis of crustaceans by interacting with retinoid X receptor (RXR) and crustacean hyperglycemic hormone (CHH) signaling. Moreover, it can also interact with other nuclear receptors, disrupting methyl farnesoate and ecdysteroid signaling, thereby altering growth and sexual maturity, respectively. This compound also interferes in cytochrome P450 system disrupting steroid synthesis and reproduction. Crustaceans are also important fisheries worldwide, and its consumption can pose risks to human health. However, some questions remain unanswered. This mini review aims to update information about the effects of OTs on the metabolism, growth, and reproduction of crustaceans; to compare with known effects in mammals; and to point aspects that still needs to be addressed in future studies. Since both macrocrustaceans and microcrustaceans are good models to study the effects of sublethal TBT contamination, novel studies should be developed using multibiomarkers and omics technology.

## Introduction

Organotins (OTs) are organometallic compounds in which an atom of tin (Sn) is covalently bounded to one or more organic chains (1). They are considered some of the most toxic chemicals introduced into aquatic environments by anthropogenic activities (1–3). OT’s deleterious effects upon wildlife and experimental animals are well documented and include endocrine disruption, immunotoxicity, neurotoxicity, genotoxicity, and metabolic dysfunction including obesity (2, 4). Butyltins (BTs) and phenyltins, the major species of OTs, are widely used for agricultural purposes (insecticides, fungicides), in PVC industry, as industrial catalysts, and as additives on boat hull’s paints to avoid encrustations by barnacles, mussels, algae, and other aquatic invertebrates (1–3, 5, 6). Therefore, large quantities of OTs have been released into aquatic ecosystems, either directly as wastewater treatment plants or indirectly as hull’s residues, posing serious environmental risks to non-target species (5, 6). Even though the use of OTs was banned in 2008, as determined by the International Marine Organization in 2001 (7), high levels of OTs can still be detected in different matrices such as surface water, clays, quartz, amorphous silica, natural soils, sediments, and organisms (5, 6, 8–10). OT levels vary in the different matrices and in different geographical regions, since environmental factors (e.g., pH, salinity, temperature) as well as the properties of the matrices can affect their adsorption (5). Recent studies in Europe revealed that OTs are still being released into the environment as outgoing water from boat wash pads, historic paint layers of hulls, and abandoned boats (11).

Marine sediment invertebrates, such as mollusks, ascidians, and crustaceans, can accumulate OTs (6, 8, 12–15). Since mollusks and crustaceans are important fisheries worldwide, many studies on OT accumulation and toxicity were developed in these animals (16, 17). Marine bivalves (mussels, clams, and oysters) tend to accumulate higher OT levels than fishes or crustaceans (13, 14, 16). Tributyltin (TBT) and triphenyltin, the most toxic forms of OTs, are well-recognized endocrine-disrupting chemicals of mollusks causing imposex or masculinization of females in more than 200 species (4, 13, 18, 19). Fishes and marine mammals can be contaminated either by drinking or by ingesting OTs-contaminated invertebrates. Therefore, the consumption of contaminated seafood (fishes, clams, mussels, oysters, crabs, and shrimps) can pose risks to human health (4, 6, 12, 20–22).

Crustaceans form a large and diverse clade of arthropods, whose members are usually free-living aquatic animals, with some terrestrial (isopods), parasitic (fish lice, tongue worms), and sessile (barnacles) species (17, 23, 24). Small crustacean species or microcrustaceans (water flees, brine shrimps, and copepods) and larval forms of larger species of decapods (crabs, lobsters) are major constituents of the zooplankton and have a vital role in the trophic transfer of nutrients and xenobiotics (17, 22, 25, 26). Decapod crustaceans, important worldwide fisheries, are usually marine, with few freshwater (crayfishes) and terrestrial (land crabs) species (17). Since decapods live on the sea floor, they can accumulate OTs dissolved in the water, in their food, or on the sediment (8, 27, 28). However, there is still little information about the mechanisms of OTs’ effects in crustaceans. This mini review aims to update information about the effects of OTs on the metabolism, growth, and reproduction of crustaceans; to compare with known effects in mammals, and to point aspects that still needs to be addressed in future studies.

## OTs Effects on the Metabolism

**Figure 1:**
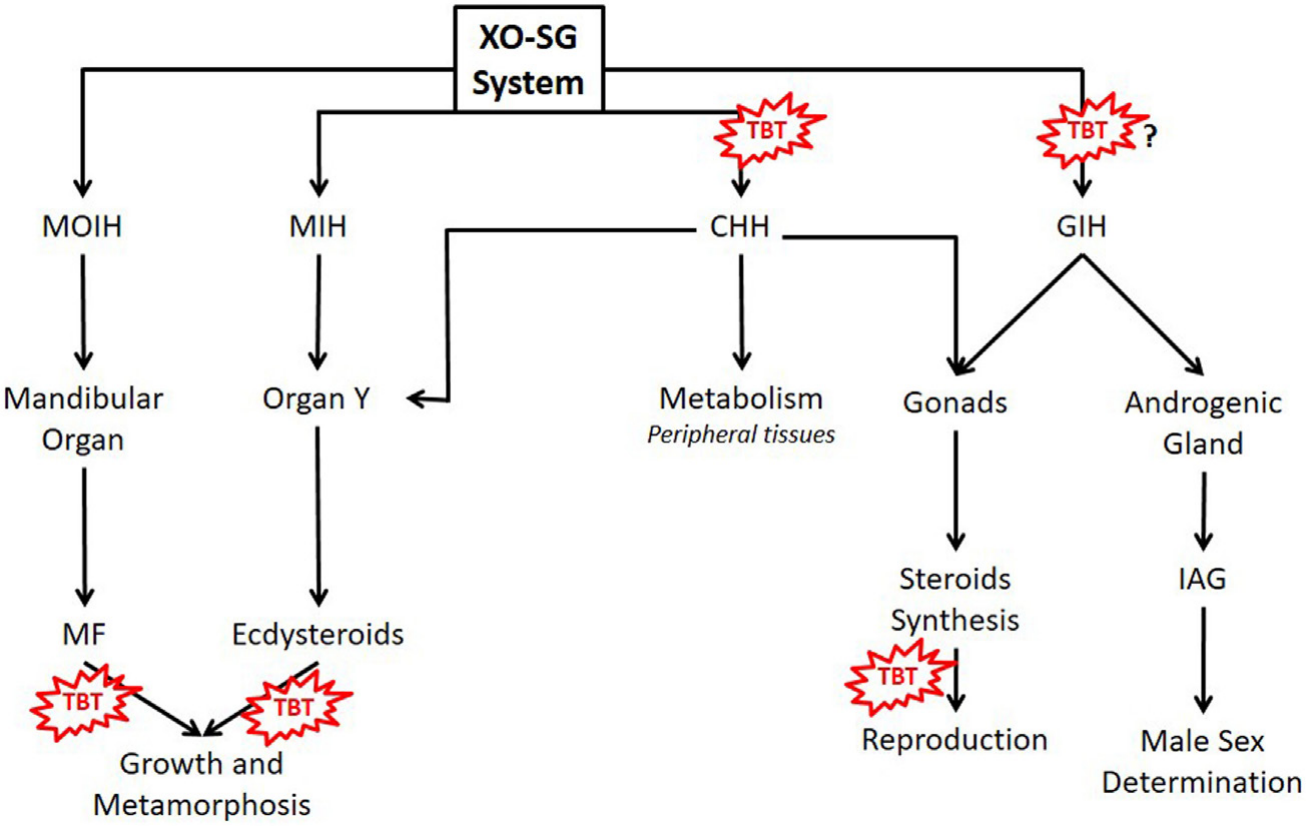
Main hormones controlling metabolism, growth, and reproduction of crustaceans and possible TBT’s action sites. MF, methyl farnesoate; MIH, molt-inhibiting hormone; MOIH, mandibular organ inhibitory hormone; CHH, crustacean hyperglycemic hormone; GIH, gonad-inhibiting hormone; IAG, insulin-like androgenic gland hormone; TBT, tributyltin; XO-SG, X organ–sinus gland system.

The main neuroendocrine center of crustaceans is the X organ–sinus gland system, located inside decapods’ eyestalk (Figure 1) (29, 30). This system is the functional counterpart of the vertebrate hypothalamus–pituitary axis, controlling many processes such as metabolism, growth, color, and reproduction (17, 29, 31, 32). It secretes neuropeptides, amines (serotonin, melatonin, and catecholamines), and opioids (enkephalins) (29, 32, 33). The most abundant neuropeptide is crustacean hyperglycemic hormone (CHH), which forms a protein family with gonad-inhibiting hormone (GIH), molt-inhibiting hormone (MIH), and mandibular organ-inhibiting hormone (MOIH). As vertebrate pituitary trophic hormones, these neuropeptides regulate other endocrine glands: gonads, androgenic gland, mandibular organ (MO), and Y organ, controlling the synthesis and secretion of other hormones (29, 32, 34).

Both macrocrustaceans and microcrustaceans are considered good animal models to study xenobiotics’ ecological and toxicological effects (16, 25, 26, 35–37). Acute toxicity assays of xenobiotics, useful to assess environmental risks, usually evaluate endpoints parameters such as mortality, egg hatching, development, growth, and reproduction (16, 25, 37, 38). These endpoints are usually expressed as median-lethal or median-effect concentrations (LC_50_ and EC_50_) and no-observed-effect-level, which can be compared with predicted environmental concentrations in exposure media for purposes of risk assessment (17, 19, 39). Decapod crustaceans exhibit higher LC_50_ values to TBT than mysidacid shrimps, copepods, amphipods, and branchiopods (16, 26, 35, 40). This higher tolerance to TBT of decapods can be related to a faster rate of TBT elimination and/or activation (16). However, larval forms of decapods are highly sensitive to TBT (41). The LC_50_ for TBT of the shrimp *Penaeus japonicus* increased progressively during initial larval stages (nauplius to mysis) and sharply after metamorphosis (41). When the larvae were exposed to hyperosmotic or hypo-osmotic stress, the osmoregulatory capacity was compromised by TBT (41).

**Figure 2:**
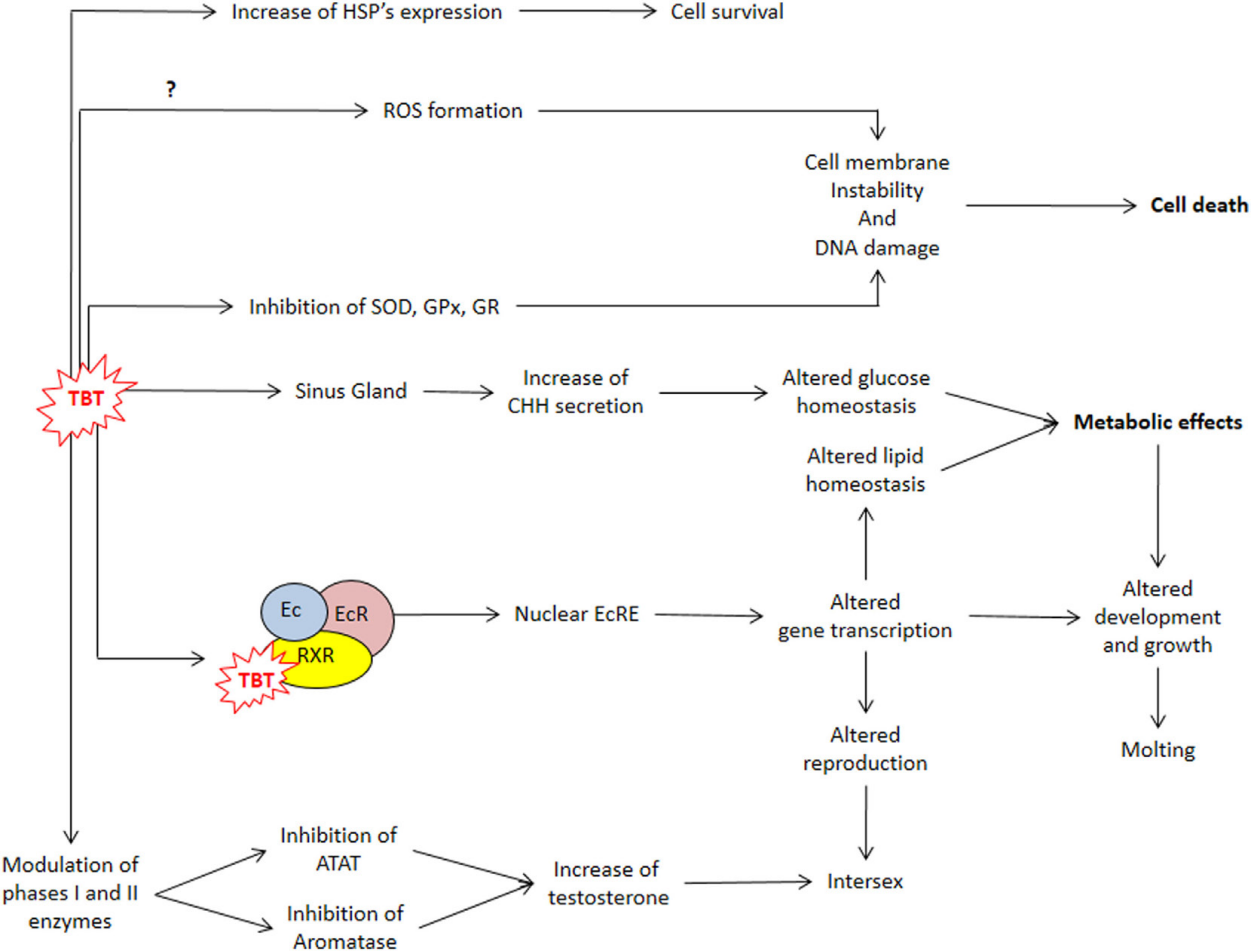
TBT actions impairing metabolism, cell protection, and reproduction. ATAT, acyl-CoA:testosterone acyltransferase; CHH, crustacean hyperglycemic hormone; Ec, ecdysteroid; EcR, ecdysteroid receptor; EcRE, ecdysteroid responsive element; GPx, glutathione peroxidase; GR, glutathione reductase; *HSP*, heat shock protein gene; ROS, reactive oxygen species; RXR, retinoid X receptor; SOD, superoxide dismutase; TBT, tributyltin.

Organotins can enter crustacean’s hemolymph from water, sediment, or food *via* gills and stomach (28, 42). Once inside the animal, their fate depends on the processes of accumulation, biotransformation (metabolism), and elimination (16, 28, 42, 43). In the hermit crab *Clibanarius vittatus*, assimilation of a single dose of TBT from food was higher than from water, and the levels of TBT in the tissues decreased progressively after 15 days, reaching null values after 75 days (44). In this study, dibutyltin (DBT) was also detected indicating an active metabolism of TBT (44). The hepatopancreas of crustaceans is an important metabolic organ that accumulates functions equivalent to vertebrate pancreas and liver: digestive enzyme synthesis, uptake and storage of nutrients, and xenobiotic’s metabolism (42, 45–49). According to their physicochemical properties, xenobiotics can be metabolized in two distinct phases: phase I—oxidation, reduction, and hydrolysis of the substance by the cytochrome P-450 (CYP) system family of proteins; and phase II—conjugation of polar groups to become soluble (28, 42, 50). Crustaceans’ hepatopancreas have an active CYP-dependent monoxygenase system that oxidizes TBT to a series of hydroxylated derivatives that are dealkylated to form DBT and/or monobutyltin (MBT) (42, 50–53). When blue crabs *Callinectes sapidus* were fed with TBT-contaminated food, TBT levels in the whole abdomen peaked to 0.12 µg g^−1^ after 4 days of feeding, while DBT and MBT peaked to 0.39 and 0.35 µg g^−1^ after 8 and 12 days of feeding, respectively (54). In another study in which *C. sapidus* were fed TBT-contaminated food, TBT levels were higher in hepatopancreas compared to gills and muscle (43). In a third study in which *C. sapidus* was fed TBT-contaminated food, the respiration rate, the expression of P-450 3A (CYP3A), and heat shock proteins (HSPs) in the hepatopancreas increased, indicating that the crabs were stressed by TBT (51). An active heat shock response, specially with increased HSP70 expression, occurs when crustaceans are exposed to many types of environmental stress such as heat (55–58), metals (59, 60), and salinity alterations (61, 62). Therefore, increased expression of HSPs could be a useful indicator of BTs/TBT contamination that should be studied in other crustacean species (Figure 2).

Reactive oxygen species (ROS), byproducts of cellular respiratory chain, are kept at physiological levels by a balance between oxidant and antioxidant agents (63, 64). Liver phase I metabolism also generates ROS as byproducts, leading to oxidative stress (OS) (37). Many drugs, pesticides, and metals induce OS in crustaceans, either by altering the expression and activity of antioxidant enzymes such as catalase, superoxide dismutase (SOD), and glutathione peroxidase (GPx) or by decreasing non-enzymatic antioxidants such as glutathione (37, 65, 66). In mammals, BTs increase ROS by decreasing the concentration and activity of SOD, GPx, and glutathione reductase (GR), while simultaneously increasing lipid peroxidation in liver, testis, and kidney (67). Since decapod crustaceans, such as the green crab *Carcinus maenas, C. sapidus*, and *Macrobrachium rosenbergii*, are considered good sentinel species, OS biomarkers should be monitored in bioassays with sublethal concentrations of BTs.

Stressed animals usually develop hyperglycemia. In vertebrates, it is considered a secondary response to the increase in catecholamine and corticosteroids’ blood levels (68, 69). In crustaceans, the main hormone responsible for triggering hyperglycemia during stress is CHH (29, 34, 70, 71). Injection of 10 μmoles of tripalmitin, fentin, and fenbutatin increased glucose levels in the hemolymph of the crab *Oziotelphusa senex senex* (72). Since this effect did not occur in the eyestalk-ablated crabs, it is possible that OTs injection caused CHH secretion (72). In *M. rosenbergii*, the treatment with TBT (10, 100, and 1000 ng L^−1^) dissolved in water for 90 days also increased glucose levels in the hemolymph (73). Therefore, synthesis, release, and secretion of CHH and its signaling are processes that could be disrupted as the result of OTs exposure and needs to be further investigated.

In mammals, TBT disrupts both glucose and lipid homeostasis: increases body weight, inflammation, adipogenesis, and blood glucose and insulin levels (2, 74, 75). These effects are mediated by alterations in insulin signaling cascade and of nuclear receptors such as estrogen receptor, peroxisome proliferator-activated receptor γ (PPARγ), and retinoid X receptor (RXR) (2, 74, 75). RXR can form both homodimers or heterodimers with many other nuclear receptors, including PPARs, and therefore bind to DNA response elements inducing the transcription of genes involved in xenoprotection, lipid homeostasis, and development (19, 76). Since TBT is recognized as a potent agonist of RXR, this binding can be considered a key step of TBT’s mechanism of action (19, 77).

The main sites of glycogen and lipid storage in decapod crustaceans are the hepatopancreas, gonads, and muscle, and these energetic reserves fluctuate in distinct species according to seasonality, reproductive stage, molt cycle, type, and regularity of the diet (46, 49, 78). These metabolites are distinctively mobilized during diverse types of stresses, reflecting homeostasis alterations that can be used as biomarkers of health and stress condition (31, 37, 46, 47, 79). In the freshwater prawn *M. rosenbergii*, TBT (10, 100, and 1,000 ng L^−1^) treatment reduced hepatosomatic index (HIS) and the content of proteins, glycogen, and lipids in the hepatopancreas in a dose-dependent manner (73). In the cladoceran *Daphnia magna*, lipids are stored in spherical lipid droplets scattered throughout the body, and treatment with 0.036 or 0.36 µg L^−1^ increased lipid fluorescent stain (80). In female *D. magna*, both doses of TBT decreased the levels of triglycerides, cholesteryl esters, and phosphocolines and increased diacylglycerol levels and altered the expression of many genes, including RXR (Figure 2) (80).

## OTs Effects on Growth

Crustacean growth, as in other ecdysozoans, occurs by the recapitulated molting process (81). Molting is regulated by a negative feedback mechanism involving CHH, MIH, and ecdysteroids (Figure 1) (81, 82). Ecdysone and 25-deoxyecdysone, inactive ecdysteroids, are secreted by the Y-organ and converted to 20-hydroxyecdysone (20-HE) and ponasterone A, the active forms, in peripheral tissues (33, 81). Ecdysteroids bind to arthropod ecdysteroid receptor (EcR) that complex with RXR (22, 80). The heterodimer EcR:RXR binds to ecdysteroid response element regulating the transcription of genes involved in development, growth, reproduction, and the genes involved in the pathways of ecdysone synthesis (17, 22, 80). Incomplete ecdysis leading to death occurs when *D. magna* is exposed to exogenous 20-HE (22). TBT alone do not alter the incidence of incomplete ecdysis; however, when in combination with 20-HE, this incidence is increased. Therefore, TBT synergizes with 20-HE leading to mortality associated with molting (22). In TBT-treated daphnids, the expression of RXR and EcR increase, disrupting the ecdysteroids’ pathways (22, 80). In the brown shrimp *Cangron cangron*, it was demonstrated that TBT fits in the ligand binding pocket of RXR, affecting the expression of RXR and EcR and probably of downstream genes (83). This genomic action of TBT was also demonstrated in the larvae of an insect *Chironomus riparius*, where TBT also increased the expression of RXR, EcR, as well as estrogen-related receptor gene and E74 (84).

Besides ecdysteroids, the sesquiterpenoids methyl farnesoate (MF) and juvenile hormone are also important during arthropod’s growth and metamorphosis (85). MF, synthesized in the MOs, is the main sesquiterpenoid of crustaceans (Figure 1) (86). The major function of MF in crustaceans is regulation of reproductive maturation (86). MF binds to methoprene-tolerant (MET), which forms a heterodimer with steroid receptor coactivator (SRC), activating the transcription of downstream genes, such as sex-determining genes involved in oocyte maturation (87). In *D. magna*, TBT also affected the expression of genes related to MF signaling pathway such as MET and SRC (80). Considering that TBT may also affect MF signaling in other crustaceans, and therefore alter their growth and development, serious impact on both planktonic and benthic communities can be expected.

## OTs Effects on Reproduction

Imposex in female gastropods is one of the better-known effects caused by TBT on invertebrates. Imposex is characterized by the formation of male sexual organs such as penis and vas deferens in these females (19, 86). Although some studies show an early sexual reversal (intersex) in crustaceans exposed to TBT, these changes are less marked than those occurring in mollusks (31, 88). Nevertheless, other detrimental effects on the reproductive system of different species of crustaceans were found in both females and males (27, 88–90). The mechanism by which TBT causes these damages is still unclear, and there are different possible sites of action (80, 86, 89).

Unlike mollusks, when female crustaceans are exposed to TBT, there is no formation of complete male sex organs (31). Nevertheless, in *M. rosenbergii*, the treatment with TBT (10, 100, and 1000 ng L^−1^) for 45 days altered ovarian morphology and induced spermatogonia and ovotestis (with spermatocytes and structures similar to seminiferous tubules) (88). In the hermit crab *C. vittatus*, TBT induced several degrees of ovarian disorganization with follicular atresia and irregular oocytes although there was no formation of male sexual structures (27). Besides damage to reproductive organs, TBT may impair reproductive rates in further generations. Juvenile female *D. magna* exposed to TBT (100 and 1,000 ng L^−1^) produced smaller newborn neonates than those of unexposed females and suffered a higher mortality during their adulthood, which resulted in lower reproductive output and fitness. The reproductive rates of exposed female’s first clutch were also lower than control (80).

Although the main described effect of TBT is the masculinization of females, it also causes damage to male reproductive organs. In *M. rosenbergii*, exposure to TBT (10, 100, and 1,000 ng L^−1^) for 45 or 90 days caused several damages to the gametes and to the gonadal tissue itself. The gonadosomatic index of the testes reduced, and the seminiferous tubules architecture was compromised by an increase in connective tissue and immature cells (spermatogonia and spermatocytes) (73, 90). Spermatozoa count and length reduced (73, 90). The activity of the antioxidant enzymes SOD, GPx, and GR reduced in the testes, while DNA damage increased (89). These results are in line with studies in mammals such as the hamster *Mesocricetus auratus*, where TBT also caused alterations in testicular histology and reduction in spermatogenesis and in enzymatic and non-enzymatic antioxidants (67).

Since sex steroids are the major regulators of vertebrate reproduction, many steroidogenic enzymes and steroid receptors seem to have co-evolved (91, 92). However, the role of vertebrate-type sex steroids on invertebrate reproduction is not well determined (19). In mollusks, TBT-induced imposex correlates with increased free testosterone (T) levels, probably induced by inhibition of acyl-CoA:testosterone acyltransferase, which conjugates T with fatty acids, and/or CYPs, reducing T clearance (19, 93). The stimulatory effects of steroids on crustacean reproduction are well recognized; however, it was only with the development of modern omics technology that genes of steroidogenic enzymes and putative steroid receptors were identified (31, 39, 94–98). In female *M. rosenbergii*, TBT reduced 17β-estradiol in the hemolymph and ovary and increased T levels in the ovary (88), while in males, TBT reduced T levels in testis (73, 90) (Figure 2) (53, 94). In crustaceans, an alternative action proposed was that TBT could block T excretion, but results are still inconclusive (18, 93, 99, 100).

The synthesis and release of steroids in crustaceans is controlled mainly by GIH and CHH, released from the ES-SG system (Figure 1) (32, 39). As already mentioned, OTs can stimulate CHH release and probably also interfere with other peptides of the CHH family such as GIH (72). Gonad-stimulating hormone, released from the brain and thoracic ganglion, monoamines, and MF also participate in the control of crustacean reproduction (32, 33, 39). GIH and MIH also regulate a peptide hormone called insulin-like androgenic gland hormone, synthesized by the androgenic gland, which is responsible for male sexual differentiation (39, 97). Therefore, there are many sites where TBT may affect the neuroendocrine regulation of crustacean’s reproduction.

## Conclusion

Crustaceans form a large group of aquatic animals that are important from both the economic and the ecological perspectives. They are important members of zooplankton and benthic communities and have vital roles in food chains, so the endocrine-disrupting effects of TBT on crustaceans can affect other organisms. They are also important fisheries worldwide. Therefore, human consumption of TBT-contaminated crustaceans can pose risks to human health. In summary, TBT can disrupt carbohydrate and lipid homeostasis of crustaceans by interacting with RXR and CHH signaling and can interact with other nuclear receptors, such as EcR, MET, and SRC, disrupting MF and ecdysteroid signaling, thereby altering growth and sexual maturity, respectively. This compound also interferes in cytochrome P450 system disrupting steroid synthesis and reproduction. Both macrocrustaceans and microcrustaceans are good models to study the effects of sublethal TBT contamination, usually found in natural environments. Multibiomarkers studies focusing on TBT’s effects on molecular, biochemical, cellular, morphological, physiological, and behavioral endpoints can be developed with crustaceans. The recent advances in omics technology, with the development of transcriptomes, lipidomes, and proteomes, are providing a novel set of information. The knowledge of the genes involved in the growth, development, and reproduction of crustaceans will certainly provide novel insights about TBT effects.

## Author Contributions

EV wrote Sections “Introduction,” “OTs Effects on the Metabolism,” and “OTs Effects on Growth.” JM wrote Sections “OTs Effects on Reproduction” and “Conclusion” and elaborated figures. AV reviewed the manuscript.

## Conflict of Interest Statement

There is no potential conflict of interest including any financial, personal or other relationships with other people or organizations related to this manuscript. The reviewer EV and handling Editor declared their shared affiliation.
